# Effect of Human Adipose Tissue Mesenchymal Stem Cells on the Regeneration of Ovine Articular Cartilage

**DOI:** 10.3390/ijms161125989

**Published:** 2015-11-09

**Authors:** Alessandro R. Zorzi, Eliane M. I. Amstalden, Ana Maria G. Plepis, Virginia C. A. Martins, Mario Ferretti, Eliane Antonioli, Adriana S. S. Duarte, Angela C. M. Luzo, João B. Miranda

**Affiliations:** 1 Department of Orthopedic Surgery, Faculty of Medical Sciences, Universidade de Campinas (UNICAMP), Campinas/SP 13083-887, Brazil; jotamiran@yahoo.com.br; 2 Hospital Israelita Albert Einstein, São Paulo/SP 05652-900, Brazil; mario.ferretti@einstein.br (M.F.); eliane.antonioli@einstein.br (E.A.); 3 Department of Pathology, Faculty of Medical Sciences, Universidade de Campinas (UNICAMP), Campinas/SP 13083-887, Brazil; ingrid@fcm.unicamp.br; 4 Instituto de Química de São Carlos, Universidade de São Paulo (USP), São Carlos/SP 13566-590, Brazil; amplepis@iqsc.usp.br (A.M.G.P.); virginia@iqsc.usp.br (V.C.A.M.); 5 Hematology Hemotherapy Centre, Universidade de Campinas (UNICAMP), Campinas/SP 13083-878, Brazil; adrianassduarte@uol.com.br (A.S.S.D.); angela.luzo@gmail.com (A.C.M.L.)

**Keywords:** tissue engineering, regenerative medicine, adult stem cell, stem cell therapy, biomaterials, cartilage

## Abstract

Cell therapy is a promising approach to improve cartilage healing. Adipose tissue is an abundant and readily accessible cell source. Previous studies have demonstrated good cartilage repair results with adipose tissue mesenchymal stem cells in small animal experiments. This study aimed to examine these cells in a large animal model. Thirty knees of adult sheep were randomly allocated to three treatment groups: CELLS (scaffold seeded with human adipose tissue mesenchymal stem cells), SCAFFOLD (scaffold without cells), or EMPTY (untreated lesions). A partial thickness defect was created in the medial femoral condyle. After six months, the knees were examined according to an adaptation of the International Cartilage Repair Society (ICRS 1) score, in addition to a new Partial Thickness Model scale and the ICRS macroscopic score. All of the animals completed the follow-up period. The CELLS group presented with the highest ICRS 1 score (8.3 ± 3.1), followed by the SCAFFOLD group (5.6 ± 2.2) and the EMPTY group (5.2 ± 2.4) (*p* = 0.033). Other scores were not significantly different. These results suggest that human adipose tissue mesenchymal stem cells promoted satisfactory cartilage repair in the ovine model.

## 1. Introduction

Articular cartilage is a specialized aneural, avascular and alymphatic connective tissue of mesodermal origin that covers the articulating ends of diarthrodial joints [1]. It is a permanent type of hyaline cartilage. Articular cartilage is significantly different in terms of structure, extracellular matrix, gene expression profile and mechanical properties than transient hyaline cartilage, which is found in growth plates and suffers ossification over time [2]. Cartilage injuries of the knee joint have been increasingly diagnosed, particularly in young active people. An estimated 61% of patients who undergo knee arthroscopy have a chondral lesion [3,4,5]. The vast majority are partial thickness lesions (95%), which preserves the calcified layer and subchondral bone [6]. In some cases, these lesions can cause symptoms such as pain and joint swelling, and they are considered to be risk factors for osteoarthritis development [6,7]. Durable restoration of damaged articular cartilage is a valuable but still unachieved goal, as reduced vascularity, limited cell populations and dense extracellular matrix impair regeneration [8]. Recent data suggest that cell therapy, based on chondrocyte implantation, is the current gold standard therapy for treating chondral lesions in the clinical setting [9,10,11]. However, the drawbacks associated with the use of adult autologous chondrocytes include the requirement for a two-step surgery; donor site morbidity; the small quantity of cells available, thus requiring a long cell culture time before implantation; and the occurrence of cellular senescence and dedifferentiation [10]. To overcome these problems, researchers are now focused on the use of advanced biological tissue engineering techniques utilizing different cell sources.

Mesenchymal stem cells (MSCs) have been frequently used to treat chondral lesions in the experimental setting. Small and large animal studies have demonstrated the utility of cartilage tissue regeneration in lesions via the implantation of constructs derived from MSCs seeded in biomaterials, with or without growth factors [11,12,13]. Tissue reconstruction by cell-based strategies could be accomplished by two mechanisms: differentiation of the implanted cells (progeny) or stimulation of endogenous healing by trophic activity (paracrine activity). MSCs can be obtained from different sources. Bone marrow-derived MSCs (BM-MSCs) are currently the most frequently used cell source. However, harvesting bone marrow is a painful and inefficient procedure because BM-MSCs constitute only the 0.002% of the material within the stromal cell population [14].

Since the report of the presence of multipotent stem cells within the heterogeneous cell population in the stromal vascular fraction of lipoaspirate by Zuk in 2001 [15], adipose tissue mesenchymal stem cells (AT-MSCs) have received more attention. Compared to bone marrow, adipose tissue is abundant and easily accessed within the body [16]. AT-MSCs constitute up to the 2% of the material within the stromal vascular fraction, and the number of cells in an equal volume of adipose tissue exceeds that in bone marrow aspirate by approximately 300-fold [17]. Due to differences in growth kinetics, signs of senescence occur later in AT-MSCs compared to BM-MSCs [17]. Like other MSCs, AT-MSCs have immunosuppressive capability, and can be xenogeneically transplanted without the need of immunosuppressant drug administration [18].

Transplantation of xenogeneic human AT-MSCs has not been fully investigated. It has only been tested in small animals [18,19]. Successful therapies in pre-clinical large animal models are required before progressing to human studies. The aim of our study was to evaluate the efficacy of human undifferentiated AT-MSCs embedded in a collagen/chitosan scaffold in healing a partial thickness lesion in the hyaline articular cartilage of a large animal model. The novelty of this study is that a lot of BM-MSCs large animal results have been reported [11], but to the best of our knowledge, this is the first study to report the positive results of using AT-MSCs to treat partial thickness chondral lesions in a large animal model.

## 2. Results

### 2.1. MSC Characterization and Differentiation

**Figure 1:**
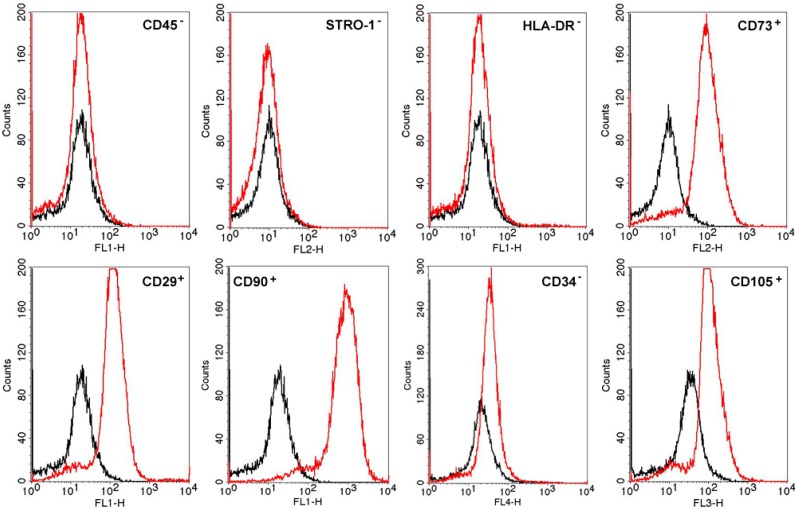
Results of flow cytometry analysis of the AT-MSCs. The cells were expanded to the fourth passage and analyzed by flow cytometry (10,000 cells were analyzed). Black histogram represents the expression of each molecule of IgG (negative control) and red histogram shows different markers investigated in the cell membrane of AT-MSCs.

Flow cytometry results showed that the isolated AT-MSCs exhibited around 95% positive staining for CD90, CD105, CD73 and CD29 and less than 2% positive staining for HLA-DR, CD45, CD34 and STRO-1 (Figure 1).

**Figure 2:**
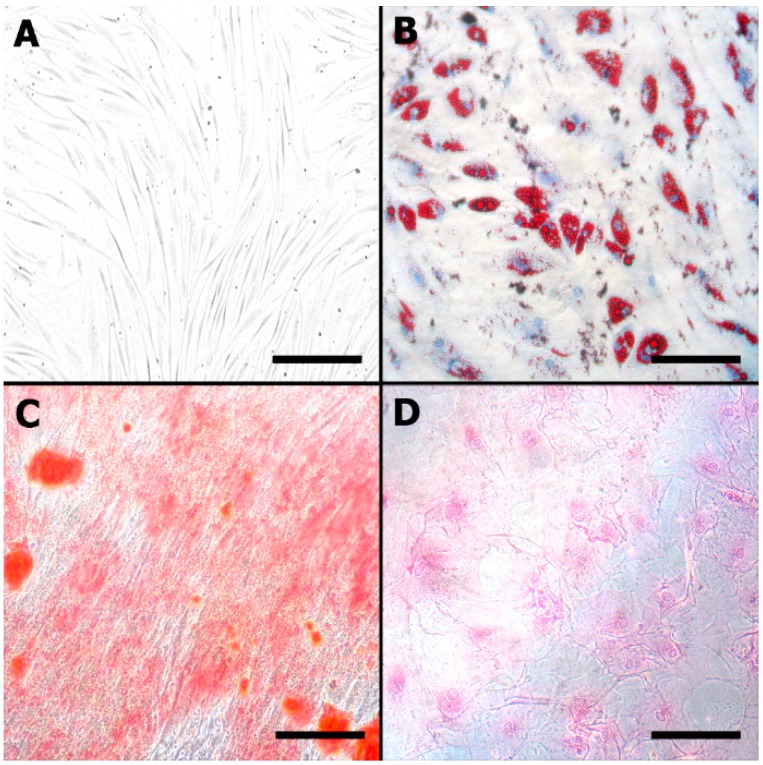
Characterization of the isolated AT-MSCs (objective, ×10): (**A**) Spindle-shaped morphology on light microscopy; (**B**) AT-MSC differentiation into the adipogenic cell lineage (Oil Red O); (**C**) osteogenic cell lineage (Alizarin red); and (**D**) chondrogenic cell lineage (Sirius red, resorcin and fuchsine). Scale bars represent 100 μm.

The adherent cells acquired a spindle-shaped morphology that was confirmed by light microscopy (Figure 2). The results of cell cultures submitted to classic differentiation protocols demonstrated that AT-MSCs were multipotent and differentiated into the adipogenic, osteogenic and chondrogenic mesodermal lineages (Figure 2).

### 2.2. Viability Assay

**Figure 3:**
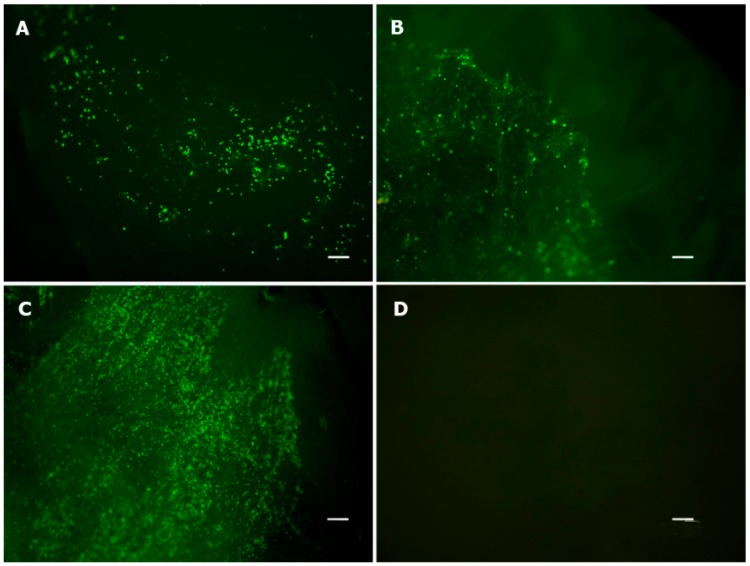
Cell viability assay. Live/Dead Viability/Cytotoxicity Kit for mammalian cells (Invitrogen, Carlsbad, CA, USA). (**A**) One-day cell culture; (**B**) three-day cell culture; (**C**) seven-day cell culture; and (**D**) control scaffold without cells to exclude natural collagen fluorescence. Live cells are green (objective, ×4). Scale bars represent 200 μm.

**Figure 4:**
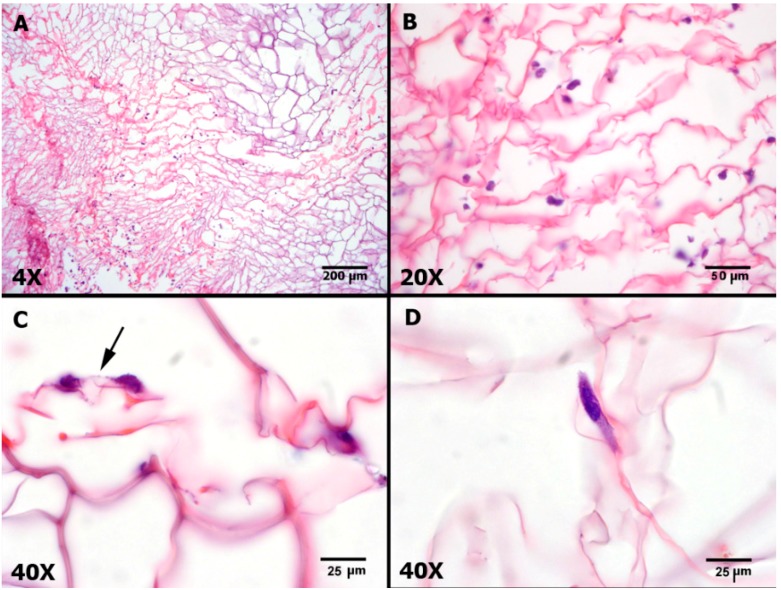
Hematoxylin and Eosin (H&E) stained histological sections of the chitosan/collagen scaffold seeded with AT-MSCs after seven days in culture. (**A**,**B**) Panoramic view of AT-MSCs uniform distribution into the scaffold; (**C**) Arrow indicates the interaction of two cells on the scaffold; (**D**) Detail of AT-MSC adhered on the scaffold.

A live/dead assay and a conventional light microscopy analysis demonstrated that the collagen/chitosan scaffold used in this study was suitable for adhesion, spreading and maintenance of AT-MSCs (Figure 3). The cells were able to penetrate into the scaffolds (Figure 4), and they remained viable after seven days in culture (Figure 3C).

This result confirmed that our collagen/chitosan scaffold promoted an adequate microenvironment to support AT-MSC survival, attachment and migration. However, the live/dead ratio was not statistically analyzed because the sample size was too small (*n* = 3) and because almost all cells were alive after seven days in cell culture (Figure 3C). As shown in Figure 3D, no red or green fluorescence was observed in an acellular scaffold, which excludes the possibility that the above results were confounded by collagen autofluorescence.

Histological sections illustrated the close interaction between the seeded AT-MSCs and collagen/chitosan scaffold after seven days in culture (Figure 4). AT-MSCs adhered to the porous structure of the scaffold, suggesting a possible reorganization of the microenvironment to enable cell proliferation and extracellular matrix synthesis.

### 2.3. Animal Experiment

**Figure 5:**
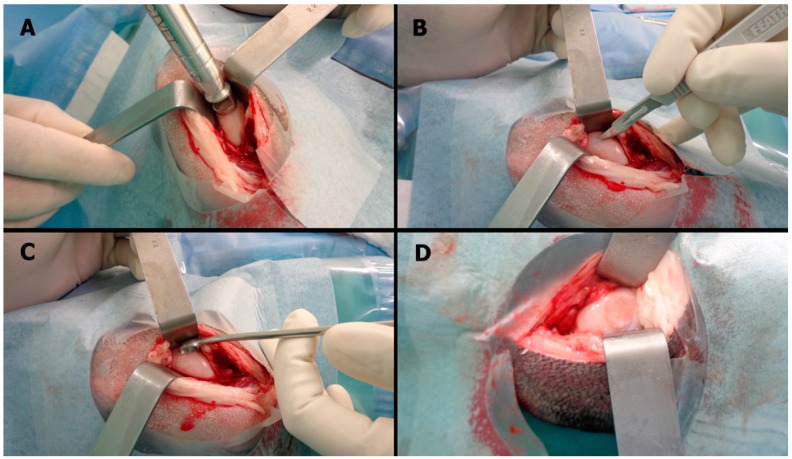
Surgical technique: (**A**) A ten-millimeter punch biopsy was used to demarcate a cylinder in the medial femoral condyle; (**B**) a number 11 blade scalpel was used to make serial parallel incisions in the cartilage surface followed by a new series of parallel incisions perpendicular to the first ones; (**C**) a surgical curette was used to remove the incised cartilage without evoking bleeding at the base of the lesion; and (**D**) the scaffold was fixed with 1 mL of fibrin glue for five minutes, and the stability was then checked with ten cycles of passive full range of motion.

All of the animals completed the six-month follow-up period. None presented with major complications, such as infection or permanent limping. The mean weight in each group was as follows: CELLS = 52.9 ± 8.7 kg, SCAFFOLD = 58.0 ± 13.2 kg, and EMPTY = 63.2 ± 10.5 kg (*p* = 0.999). Figure 5 presents the surgical steps involved in creating the lesion and implanting the scaffold.

### 2.4. Macroscopic Findings

**Figure 6:**
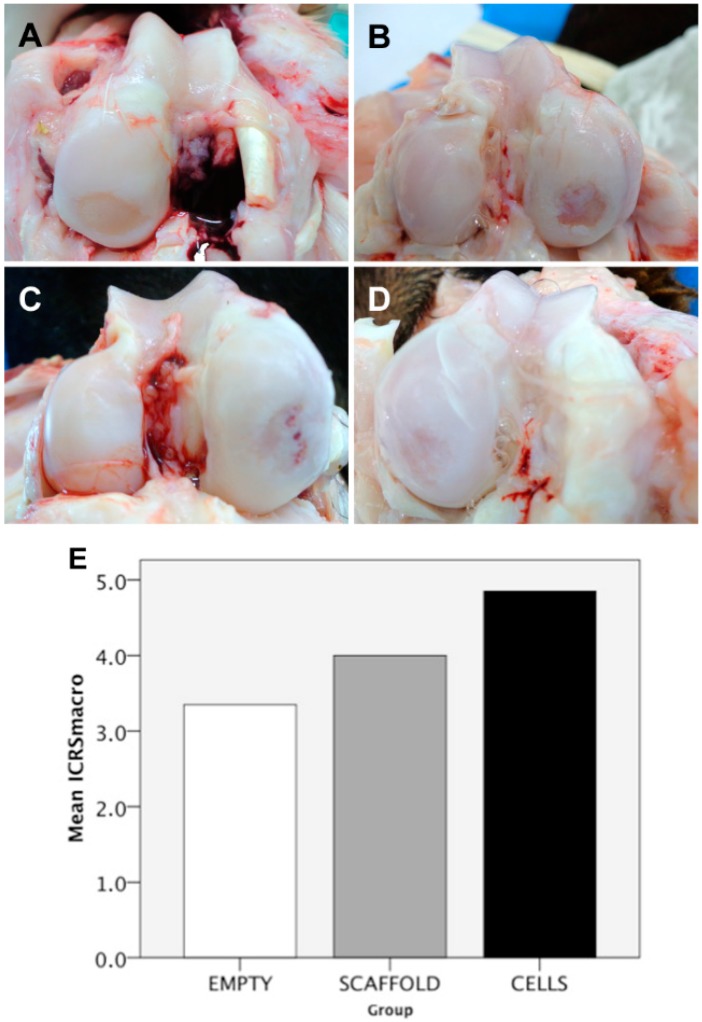
Macroscopic results: (**A**) Completely empty defect (group EMPTY); (**B**) partially filled defect (group EMPTY); (**C**) completely filled defect, but with the presence of a gross vascularized tissue (group SCAFFOLD); (**D**) completely filled defect (group CELLS); and (**E**) International Cartilage Repair Society (ICRS) macroscopic score results presented as a bar chart (EMPTY = 3.4 ± 3.3; SCAFFOLD = 4.0 ± 2.2; CELLS = 4.9 ± 2.2; *p* = 0.738).

The mean ICRS macroscopic score was 4.9 ± 2.2 in the CELLS group, 4.0 ± 2.2 in the SCAFFOLD group and 3.4 ± 3.3 in the EMPTY group (*p* = 0.738) (Figure 6). This score ranges from 0 to 12. The higher the score, the better the outcome. Three parameters are evaluated: degree of defect repair; integration to the borders; and macroscopic appearance. Despite this semi-quantitative score have found no difference between groups, a subjective observation showed that among the defects in the CELLS group (*n* = 10), three were completely empty, four were partially filled, and three were completely filled. In the SCAFFOLD group (*n* = 10), three defects were completely empty, six defects were partially filled, and only one defect was completely filled. In the EMPTY group (*n* = 10), five defects were completely empty, and five defects were partially filled; none were completely filled. At the time of harvest, no free bodies, infections or signs of synovitis were observed. No macroscopic changes at the opposite tibial surface were observed.

### 2.5. Microscopic Findings

**Figure 7:**
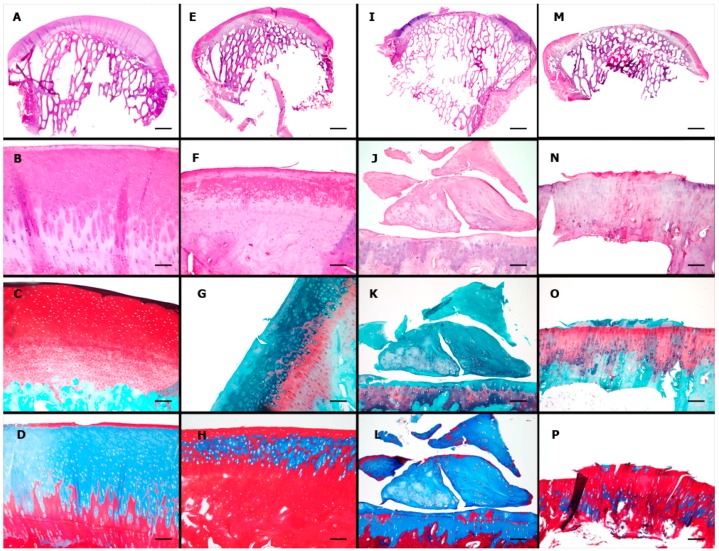
Microscopic results: first row, panoramic view; second row, H&E stain; third row, Safranin O (used to color in red the glycosaminoglycans); and fourth row, Masson’s trichrome (used to color in blue the collagens and new connective tissues). (**A**) Panoramic view by scanning; scale bar = 2 mm; **(B**–**D**) objective, ×10; scale bars = 100 μm: normal ovine cartilage; (**E**) scanning; scale bar = 2 mm; (**F**–**H**) objective, ×10; scale bars = 100 μm: group CELLS; a smooth continuous surface with complete horizontal filling and complete integration to the borders, but partial vertical filling of the defect and an heterogeneous Safranin O staining of the extracellular matrix; (**I**) scanning; scale bar = 2 mm; (**J**–**L**) objective, ×10; scale bars = 100 μm: group SCAFFOLD; defect partially filled with fibrocartilage; (**M**) scanning; scale bar = 2mm; and (**N**–**P**) objective, ×10: group EMPTY; defect unfilled. Scale bar = 100 μm.

Substantial variability was also observed in the histological examinations, especially in the EMPTY group. Examples of the normal aspect of the ovine cartilage and representative results of each group are illustrated in Figure 7.

**Figure 8:**
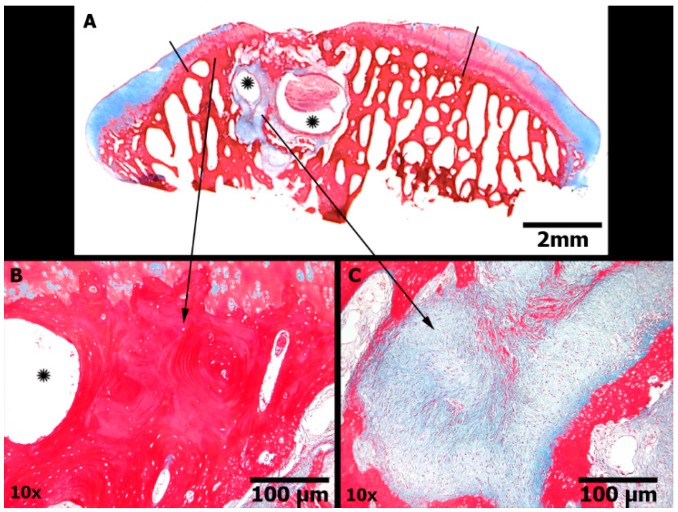
Subchondral bone damage (Masson’s trichrome stain). Group SCAFFOLD. (**A**) Panoramic view of the articular surface in the region of the defect (delimited by black lines) showing cartilage border centripetal ingrowth. Evident cystic formations were noted (asterisk) in a localized area of subchondral bone, surrounded by loosely immature fibrous connective tissue and new sclerotic bone, similar to the findings of advanced degenerative joint disease; Scale bars represent 2 mm; (**B**) Sclerotic bone; Scale bars represent 100 μm; (**C**) Immature fibrous tissue; Scale bars represent 100 μm.

The ICRS 1 scale is composed of six sub-items. The mean total ICRS 1 score, excluding the Calcified Cartilage sub-item, was 8.3 ± 3.1 in the CELLS group, 5.6 ± 2.2 in the SCAFFOLD group and 5.2 ± 2.4 in the EMPTY group (*p* = 0.033). The results of each sub-item are shown in Table 1. The scores for the CELLS group were only statistically superior for the sub-items Matrix and Subchondral Bone. Figure 8 illustrates the degenerative changes in the subchondral bone in a case in the SCAFFOLD group. The position of the calcified cartilage was normal in all cases because the lesion was partial thickness, and thus the calcified cartilage was preserved. Therefore, an adaptation of the ICRS 1 scale was adopted in this study, whereby the sub-item Calcified Cartilage was excluded.

To overcome some problems with the ICRS 1 and a partial thickness model, especially regarding the sub-item Calcified Cartilage, a new Partial Thickness Model scale was proposed in this study. The mean score for the new scale was 22.0 ± 3.8 in the CELLS group, 17.1 ± 3.7 in the SCAFFOLD group, and 19.7 ± 7.3 in the EMPTY group (*p* = 0.135). The results of the sub-items are presented in Table 2. This new scale is composed of seven sub-items. The scores for the CELLS group were statistically superior for the sub-items Horizontal Filling and Cellularity.

**Table 1:** Absolute frequencies of the ICRS 1 microscopic scale sub-items. Sub-item VI (Calcified Cartilage) was not used because the partial thickness model preserved this layer.

	Sub-Item	Score	CELLS (*n* =10)	SCAFFOLD (*n* = 10)	EMPTY (*n* = 10)	*p*
I	Surface	3	3	1	0	0.089
		0	7	9	10	
II	Matrix	2 or 3	5	2	0	0.013 *
		0 or 1	5	8	10	
II	Cell Distribution	2 or 3	5	3	1	0.131
		0 or 1	5	7	9	
IV	Cell Viability	3	5	2	3	0.349
		0 or 1	5	8	7	
V	Subchondral Bone	2 or 3	6	1	4	0.050 *
		0 or 1	4	9	6	

ICRS 1: International Cartilage Repair Society. * Indicates *p* < 0.05.

**Table 2:** The Partial Thickness Model scale results, expressed as mean ± S.D.

Sub-Item	CELLS (*n* = 10)	SCAFFOLD (*n* = 10)	EMPTY (*n* = 10)	*p*
Horizontal filling	2.0 ± 0.7	1.8 ± 0.6	1.3 ± 0.5	0.044 *
Vertical filling	1.6 ± 0.7	1.6 ± 0.7	1.4 ± 0.8	0.541
Cellularity	1.3 ± 0.5	1.0 ± 0.5	0.7 ± 0.5	0.038 *
Safranin	0.7 ± 0.5	0.7 ± 0.5	0.6 ± 0.7	0.801
Borders	1.9 ± 0.3	1.6 ± 0.5	1.6 ± 0.5	0.251
Residual calcified cartilage	2.2 ± 0.9	1.4 ± 0.8	2.1 ± 1.1	0.132
Subchondral bone	3.0 ± 0.7	2.3 ± 0.7	3.1 ± 1.0	0.090
Total	12.7 ± 2.2	10.5 ± 2.1	11.2 ± 3.6	0.167

* Indicates *p* < 0.05.

## 3. Discussion

The treatment of focal chondral lesions remains a challenge for medical science. Numerous pharmacological and surgical treatments have failed to improve the healing capacity of this tissue. Tissue engineering presents a new hope for promoting articular cartilage regeneration based on the implantation of cells, scaffolds and growth factors.

MSCs are multipotent non-hematopoietic progenitor cells of stromal origin. With adequate growth factors treatment, they can be induced to differentiate into adipose, osteogenic and chondrogenic cell lineages. They are present within many tissues that have potential as harvest sources. Needle aspiration of bone marrow (BM) from the iliac bone has been the most used method to obtain MSCs. Synovial, periosteal and adipose tissue are other sources that have been assessed in animal experiments. Synovial-derived MSCs and BM-MSCs were shown to have superior performance compared to AT-MSCs in the production of cartilage phenotype in the *in vitro* differentiation assays [17]. However, there is evidence that MSCs pre differentiation into the chondrogenic lineage in the laboratory does not produce a permanent phenotype. Instead, they behave as transient hyaline cartilage, such as that found in growth plate chondrocytes, and express a hypertrophic phenotype, which frequently leads to degeneration or hypertrophy over time [20,21]. A less explored different point of view is that the paracrine effect of MSCs may be a mechanism more relevant than their differentiation potential in mediating osteoarticular repair [17]. The rationale behind the use of undifferentiated MSCs in our study concerns the anti-inflammatory and immunomodulatory properties of these cells, which could facilitate tissue regeneration by roaming autologous host cells and creating a propitious environment [22,23]. Cultured MSCs demonstrated the ability to secrete a wide range of proteins with anti-apoptotic and immunomodulatory effects [24]. Based on this alternative point of view, adipose tissue has become an attractive cell source. Compared to bone marrow, adipose tissue has many advantages for tissue engineering: it is abundant and easily accessed within the body [16]; the number of MSCs in the adipose tissue is much higher than BM [17]; and signs of senescence occur later in MSCs derived from adipose tissue [17]. The hypothesis of our study was that undifferentiated AT-MSCs implanted in the knee of adult sheep would enhance the regeneration of a lesion in the articular cartilage, compared to the control groups.

The results of this study indicate that undifferentiated AT-MSCs improved the quality of the repair tissue, according to the criteria of the semi quantitative ICRS 1 scale. The sub-items for which the AT-MSCs demonstrated superiority were “Matrix” and “Subchondral Bone”. Although there were not any cases with a true characteristic hyaline matrix in any group, the CELL group most frequently exhibited mixed hyaline and fibrocartilage tissue, whereas the defects in the SCAFFOLD and EMPTY groups were basically filled with fibrocartilage. Degenerative changes in the subchondral bone were more evident in the SCAFFOLD group than in the CELLS group. Interestingly, the EMPTY group showed subchondral bone similar to that in the CELLS group, indicating that mechanical overload is probably not the only mechanism responsible for bone damage. We hypothesize that the fibrin glue or the scaffold itself might have some inflammatory effects at the base of the lesion that were avoided by the presence of the paracrine effect of the AT-MSCs. The fibrin glue and the scaffold were from xenogeneic origin, as the cells. The use of xenogeneic cells was previously validated in this model, and it circumvents the need for two surgical procedures in the animal, thereby reducing suffering [25]. Articular cartilage does not have blood vessels, theoretically evoking immune-privileged status. Although a recent study questioned the use of xenogeneic cells by reporting active graft rejection of human chondrocytes in an animal model [26], it is important to remember that AT-MSCs have immunomodulatory and anti-inflammatory properties that are not present in chondrocytes. The results of Bordeaux *et al.* [19] and Wang *et al.* [18] support the use of xenogeneic AT-MSCs to promote articular cartilage repair. But the fibrin glue and the scaffold probably induced a local response that enhanced subchondral degenerative changes only in the SCAFFOLD group. This result could not be attributed to differences in calcified cartilage damage at the base of the lesion because the new scale specifically evaluated this feature in a sub-item, and there were no differences between the three groups. The new scale was unable to detect a difference between the groups, probably because type 2 error. The sub-items in this new scale that demonstrated superiority in the CELLS group were Horizontal Filling and Cellularity. The macroscopic findings of our study revealed that the number of lesions that were completely filled was higher in the group treated with AT-MSCs (CELLS). However, the semi-quantitative evaluation using the ICRS macroscopic scale did not show a significant difference compared to the control groups, probably because of a type 2 error. Taken together, these findings support the conclusion that the implantation of AT-MSCs improved the quality of the repair tissue compared to control groups, but are still far from the quality of the normal hyaline cartilage.

The comparison with other results in the literature is difficult because of the variability in the protocols. The use of undifferentiated MSCs was tested previously in a few studies, with discrepant results. Matsumoto *et al.* [27] used undifferentiated BM-MSCs seeded into a collagen gel to treat osteochondral lesions in the rabbit knee, but this study was unable to demonstrate a superior result compared to the control group. Wilke *et al.* [28] used undifferentiated BM-MSCs directly injected with a fibrin vehicle in full thickness defects in the knee of six young horses; MSCs improved the early healing response evaluated macroscopically by arthroscopic assessment 30 days after implantation, but did not enhance the long-term histological appearance compared to the control group after eight months, according to a self-made histological scoring system. Lim *et al.* [29] used undifferentiated BM-MSCs seeded in a synthetic hydrogel scaffold in an osteochondral model in minipigs; the assessment with the Wakitani histological score was considered superior to the control group. Regarding the use of AT-MSCs, there are a few studies with good results in small animal models. In a previous study, our group obtained significantly higher macroscopic and ICRS 1 scores with xenogeneic AT-MSCs seeded in a type 2 collagen hydrogel in a rabbit model [19]. This finding is in accordance with the recent results of Wang *et al.* [18], who described the intra-articular injection of xenogeneic human AT-MSCs in a rabbit model of osteoarthritis and demonstrated good articular cartilage repair with the modified O’Driscoll histological score. Murata *et al.* [30] described the preliminary results of a study with minipigs; they observed promising results with the implantation of autologous undifferentiated AT-MSCs for the treatment of osteochondral lesions in the knee joint. However, it is well known that the generalizability to human beings of the results obtained in small animals experiments is limited. Positive results in large animal models are essential to demonstrate efficacy of the method before the technique can be performed in humans. Bornes *et al.* [11] provided an extensive summary of the current available experiments in the literature regarding articular cartilage repair with MSCs in large animal models. According to these authors, fifteen experiments have been reported since 1994 in the PubMed, Embase and Web of Science databases. Twelve experiments used BM-MSCs, and the other three experiments used synovial-derived MSCs; none used AT-MSCs. So, to the best of our knowledge, this is the first study with the use of undifferentiated AT-MSCs in the treatment of focal chondral lesions in a large animal model.

An advantage of our study was the creation of only one defect in each knee. Certain previous studies have generated two or more defects in each knee. It is well known that cells migrate from the scaffold to the synovial fluid. Therefore, the use of a control defect in the same knee in which a cell-treated lesion is present, so as to reduce the number of animals, is inadequate. Another advantage was the creation of the defect with a scalpel and a curette, thereby avoiding lesions in the calcified cartilage. Previous studies have typically used a drill to generate the defect, which can lead to subchondral bone damage and heat production that can damage the adjacent cartilage. A drawback with the previous studies in large animals was the damage to the subchondral bone. The use of osteochondral or full thickness cartilage models, with damage to the calcified cartilage layer, allows autologous BM-MSCs to migrate to the lesion site from the bone marrow as well as blood vessel penetration into the repaired tissue. Therefore, it is not possible to ascertain whether the results are produced by the implanted cells in the scaffold or by the cells and blood vessels coming from the bone marrow at the base of the lesion. This strategy has been used for the clinical treatment of human chondral lesions (microfracture, drilling, and abrasion arthroplasty), but the repaired tissue is made of fibrocartilage. The safety of these procedures has been questioned by a recent study, because complications like bone collapse and subchondral cysts has been reported in the follow-up of patients submitted to these techniques [31]. The lesion could evolve also with the formation of intra-lesion osteophytes and could impair future corrective procedures if the first treatment fails [32,33]. Partial thickness lesions are most frequent in humans. Successful treatment of partial thickness defects without lesion of the calcified layer or the subchondral bone is important for the development of clinical strategies that could be used in humans in the future. The partial thickness model evokes an unsolved series of problems and is a challenge for researchers. This perhaps explains the discrepancies between the numerous studies using the osteochondral model while the vast majority of lesions in clinical practice are partial thickness. The first discussion point concerns scaffold fixation. The main issue is that the defect created in the sheep is typically overly shallow compared to that in humans. Fibrin glue fixation is easy to perform and is routinely used in chondrocyte implantation in humans [34]. Some authors have argued that fibrin glue has the weakest fixation strength compared to cartilage sutures, trans-osseous sutures and biodegradable pins, but it is the best way to preserve scaffold integrity [35,36]. In our study, fixation with fibrin glue was tested intra-operatively with ten cycles of passive full range of motion without implant loosening. There were no signs of loose bodies inside any joint evaluated in our study. Another issue is that the postoperative protocol in humans requires early continuous passive motion (CPM) and a period of eight to twelve weeks with crutches to avoid joint loading when fibrin glue is used to fix cell-seeded scaffolds to the articular cartilage. However, joint unloading would be a difficult task in this large animal model. Finally, evaluating the results using the ICRS 1 scale is not ideal. A major difficulty during this study was the absence of an appropriate clinical score to assess the treatment results for the partial thickness chondral lesions. The O’Driscoll score [37] is the most frequently used score in animal studies. However, the original score does not address the subchondral bone, which, from our point of view, is of great importance. Many modifications of that score (MODS) have been described, but no modification has been adapted for use in the partial thickness model. To overcome these issues, we proposed a new scoring system. Early degenerative joint disease, especially change in subchondral bone, was given a high value in this new scoring system. As this score is not yet validated, we did not use it as the primary outcome.

However, this study had some limitations. Preclinical animal model endpoints are suggested to include histology, biomechanical evaluations and safety analyses [38]. We used only macroscopic and histologic endpoints. More sophisticated histological methods, such as histomorphometry, immunohistochemical staining and polarizing light microscopy, were not performed. The histological evaluation did not show aspects of hyaline cartilage to justify the use of more sophisticated and expensive analytical methods to further characterize the repairing tissue. Another criticism was that this study histologically analyzed only one section from the central region of the defect. Ideally, more sections from the same defect would be evaluated to address heterogeneity within the repaired tissue inside the lesion.

As we cited that our group conducted a previous experiment with AT-MSCs seeded in a type 2-collagen hydrogel [19], the rationale behind the selection of the scaffold should be discussed. Chitosan/collagen type 1 scaffolds have often been used in cartilage tissue engineering [39,40,41]. Collagen, the most abundant protein in the body, has several advantages as a biomaterial, such as biocompatibility, biodegradability, low antigenicity and lack of toxicity. Another major advantage over synthetic materials is the presence of the repetitive sequence “*Arg-Gly-Asp*”, which is important for enhancing cell adhesion [42]. However, major disadvantages include the rapid degradation and poor mechanical properties of pure collagen scaffolds. That was the reason why we chose to change the collagen 2-hydrogel in the large animal experiment. We judged appropriate a material with greater resistance to the higher load and higher follow-up involved. Cross-linking and blending with other materials are efficient methods for overcoming these problems. Cross-linking can be achieved by physical or chemical methods, but physical methods cannot yield an adequate degree of cross-linking. However, chemical methods can potentially introduce toxic residuals [40]. Blending with other materials, including chitosan, seems to be a promising strategy. Chitosan is used in a variety of biomedical fields because of its hemostasis properties and ability to enhance tissue regeneration. It has an excellent ability to be processed into porous structures. Chitosan is a polysaccharide that is structurally similar to glycosaminoglycan, which has an important role in interlinking with collagen in living tissues to obtain mechanical stability and compressive strength [43,44]. However, hyaline cartilage contains collagen type 2 as the most abundant protein of the extracellular matrix. It is more difficult to obtain collagen type 2, and a blend with chitosan was not previously described; therefore, further studies are necessary before using such a blend. Another strategy to improve the scaffold is the use of jet-based technologies, like electrospinning, to create three-dimensional biological structures with regular cell distribution inside it. The traditional methods used to generate scaffolds do not control the direction of fibers. Pore sizes and shapes become irregular, making difficult the regular infiltration of manually seeded cells inside it [45]. It was demonstrated that living cells could be electrospun successfully, encapsulating the biosuspension containing the cells [46].

Finally, six months represents the most common follow-up period in large animal experiments, but it could be considered too short to evaluate degenerative changes and the behavior of the regenerative tissue. In the future, we plan to perform another study in a large animal model with human undifferentiated AT-MSCs, but with a new scaffold composed of a type 2 collagen and chitosan blend, a CPM device with a period without weight bearing and a longer follow-up time.

## 4. Experimental Section

### 4.1. Human Adipose Tissue Mesenchymal Stem Cells (hAT-MSCs)

Adipose tissue was obtained from discharged fat tissue from healthy female patients, aged 25 to 50 years, who were undergoing lipoaspiration for esthetic purposes. Patients signed a free consent form to donate the material. The study was approved by the local Ethical Committee (CEP 839/2008, Campinas, Brazil). The methods for isolating and culturing stromal stem cells from human adipose tissue have been previously described [47]. In brief, cells obtained from adipose tissue were digested with collagenase A type 1 (Gibco Invitrogen Corp, Grand Island, NY, USA), plated at a density of 1.0 × 10^6^ cells per 25-cm^2^ flask in 5 mL of low-glucose Dulbecco’s modified Eagle’s medium (DMEM; Gibco Invitrogen Corp) supplemented with fetal bovine serum (FBS; Vitrocell Embriolife, Campinas, Brazil), and incubated at 37 °C in a 90% humidity atmosphere containing 5% CO_2_ for 3 days. At 80% confluence, non-adherent cells and debris were removed, and fresh medium was added to the adherent cells. At the fourth passage, adherent cells were detached using trypsin-ethylenediamine tetra-acetic acid (EDTA).

AT-MSCs were characterized by light microscopy to evaluate morphology, by flow cytometry analysis and by differentiation into mesodermal lineages with specific protocols for the chondrogenic, adipogenic and osteogenic lineages.

#### 4.1.1. Flow Cytometry Analysis

AT-MSCs were immunophenotyped by fluorescence-activated cell sorting (FACS) (Becton Dickinson and Company, Franklin Lakes, NJ, USA). At the fourth passage, cells were detached with trypsin-EDTA, washed with phosphate-buffered saline (PBS), and immediately stained with the following labeled antibodies: CD3, CD8, CD14, CD29, CD34, CD45, CD73, CD105, CD90 and HLA-DR. Analyses were performed using the CellQuest program; 10,000 events were acquired and analyzed.

#### 4.1.2. Mesodermal Lineage Differentiation

Osteogenic differentiation was performed with fourth passage AT-MSCs cultured for three weeks in osteogenic medium: Iscove’s Modified Dulbecco Medium (IMDM) (Gibco, Grand Island, NY, USA) supplemented with 0.1 mmol/L dexamethasone (Sigma-Aldrich, Saint Louis, MO, USA), 10 mmol/l-glycerolphosphate (Sigma-Aldrich) and 0.2 mmol/L ascorbic acid (Sigma-Aldrich). The medium was changed twice weekly. Osteogenic differentiation was confirmed by light microscopy after staining the cells with Alizarin red.

Adipogenic differentiation was performed over three weeks in adipogenic medium: IMDM supplemented with 0.5 mmol/L 3-isobutyl-1-methylxanthine (Sigma-Aldrich), 1 mol/L hydrocortisone (Sigma-Aldrich), 0.1 mmol/L indomethacin (Sigma-Aldrich) and 10% rabbit serum (Sigma-Aldrich). The medium was changed twice per week. Adipogenic differentiation was confirmed by light microscopy after staining the cells with Oil Red O.

Chondrogenic differentiation was performed over 3 weeks in chondrogenic medium: high-glucose DMEM (Bio-fluid) supplemented with 0.1 mmol/L dexamethasone, 50 g/mL ascorbic acid, 100 g/mL sodium pyruvate (Sigma-Aldrich), 40 g/mL proline (Sigma-Aldrich), 10 ng/mL transforming growth factor-1 (TGF-1), 1.25 mg/mL bovine serum albumin, 5.35 mg/mL linoleic acid, and 50 mg/mL ITS (insulin, transferrin, selenium) premix (Becton Dickinson) containing 6.25 g/mL insulin, 6.25 g/mL transferrin, and 6.25 ng/mL selenious acid. Medium changes were performed twice per week. Chondrogenic differentiation was confirmed by light microscopy after staining cells with Sirius red, resorcin and fuchsine.

### 4.2. Scaffold Preparation

The method for preparing the scaffold used in this study and its chemical and mechanical properties were detailed described in previous studies [43,48].

Blended material consisted of chitosan and type 1 collagen 1:1 (*w*/*w*). The chitosan solution was slowly added to the collagen gel, and the mixture was blended with a mixer and freeze-dried to obtain cylindrical sponges with a diameter of 10 mm and a thickness of 1.5 mm. The sponges were neutralized in ammonia vapor for 48 h and then under airflow for 10 days thereafter. After this period, the material was sterilized with ethylene oxide. Three days prior to surgical implantation, 1 × 10^6^ cells (hAT-MSCs) were dropped onto the scaffold and incubated in a cell culture plate containing DMEM at 37 °C with 5% CO_2_.

### 4.3. Viability Assay

Cell viability was assessed after one, three and seven days inside the scaffold. Each cell culture experiment was conducted in triplicate. Scaffolds were stained using a Live/Dead Viability/Cytotoxicity Kit for mammalian cells (Invitrogen, Carlsbad, CA, USA) according to the manufacturer’s protocol. Control scaffolds with no cells and control cells without scaffold were also observed at each timepoint. The entire scaffold was evaluated by fluorescence microscopy using a Leica TCS SP5 II microscope (Leica Biosystems, Nussloch, Germany) equipped with a Nikon E500 camera (Nikon Corp., Tokyo, Japan). The wavelength was set at 512 nm, and images were obtained with 4× and 10× objectives immediately after fluorescent labeling. After fluorescence labeling, scaffolds were embedded in paraffin, sectioned in 5 μm slices, stained with H&E and subjected to conventional light microscopy analysis.

### 4.4. Experimental Design

All applicable international, national and institutional guidelines for the use of animals were followed. All procedures performed in studies involving animals were in accordance with the ethical standards of the Hospital Israelita Albert Einstein. Approval from the local Animal Care and Use Ethical Committee (São Paulo, Brazil) was obtained (CEUA 1556-12).

Thirty knees of fifteen Dorper sheep were randomly allocated to three groups based on a primary sample size calculation. Each animal was operated upon bilaterally. The animals were between three and five years of age. All the animals were female, and the mean weight was 58.02 ± 11.38 kg. A critical size partial thickness chondral lesion was created in the central region of the medial femoral condyle, and the groups were treated as follows:

(1) CELLS: hAT-MSCs were seeded in a collagen/chitosan scaffold that was used to fill the lesions;
(2) SCAFFOLD: the same scaffold was used to fill the lesion, but no cells were included;
(3) EMPTY: the lesion was left untreated.

The primary outcome was the ICRS 1 histological scale [49].

Secondary outcomes included a new histological scale designed to address partial thickness lesions in animal models and the ICRS macroscopic scale [50].

### 4.5. Surgical Procedure

Preoperative sedation was achieved with an intra-muscular injection of 7.5 mg/kg ketamine (Ketalar 50 mg/mL, Pfizer, São Paulo/SP, Brazil) and 0.25 mg/kg midazolam (Dormonid 5 mg/mL, Roche, São Paulo/SP, Brazil). Anesthesia was induced with a 20-mL intravenous (i.v.) injection of propofol (Diprivan 1%, AstraZeneca, Cotia/SP, Brazil). The trachea was intubated, and anesthesia was maintained with 15 mL/kg continuous inhaled isoflurane in oxygen (Forane 2%, Abbott, Buenos Aires, Argentina). Analgesia was provided by 2 mL of i.v. fentanyl (Fentanyl 0.5 mg, Janssen, São José dos Campos/SP, Brazil) and local skin infiltration with 20 mL of 2% lidocaine/1% norepinephrine solution (Xylestesin 2%, Cristalia, Itapira/SP, Brazil). The animals received a continuous infusion of Ringer’s lactate solution during all procedures. Antibiotic prophylaxis was achieved with a single dose of 2 g of i.v. cefazolin (Kefazol 1 g, ABL Antibiotics, Cosmopolis/SP, Brazil) at induction.

The animals were placed in the dorsal decubitus position on appropriate surgical beds with the knees fixed in maximal flexion to maximize exposure to the weight-bearing zone of the medial femoral condyle. The joint was opened with an anteromedial incision in standard sterile fashion without dislocating the patella. A 10-mm punch biopsy trocar was used to generate a round defect in the middle of the condyle, and a partial thickness cartilage lesion was created. The “mango technique” was used to cut out the cartilage, avoiding any bleeding at the base of the lesion [51]. The surgical steps are illustrated in Figure 5. A previous study found that defects larger than 7 mm in diameter can lead to osteoarthritis in the ovine knee model [7]. The calcified cartilage zone was left intact at the base of the lesion, thus creating a partial thickness chondral lesion without bleeding from the subchondral bone. A previous study validated that the surgical technique used in our study avoids damaging the calcified layer [52]. Implantation was conducted according to the allocation group. When scaffolds were used, they were fixed with 1 mL of fibrin glue (Tissucol, Baxter AG, Vienna, Austria). After five minutes of scaffold fixation, ten cycles of full range of motion were applied to check for implant stability. The surgical wound was closed in layers with an absorbable suture (Vicryl, Ethicon Inc., Somerville, NJ, USA), and silver spray (Bactrovet, Konig, Argentina) was used to cover the incision.

The animals were allowed to bear full weight with restricted movement in a 4 m^2^ cage for seven days postoperatively, after which the sheep were sent back to the pasture, where they were kept under veterinary care. After six months in the pasture, the animals were brought back to the center for animal research and euthanized using veterinary assistance. KCl (19.1%, 20 mL i.v. bolus) was administered to the anesthetized sheep.

### 4.6. Macroscopic Analysis

Two blinded researchers rated the quality of the newly formed tissue *in situ* at the moment of euthanasia through an extended arthrotomy, according to the ICRS macroscopic score [50]. Next, a block of the medial femoral condyle containing the entire defect and flanking the articular cartilage and subchondral bone was removed with a saw and chisel.

### 4.7. Microscopic Analysis

Blocks were fixed in 10% neutral buffered formalin for two days, washed with distilled water, decalcified in medium containing a 20% EDTA solution at pH 7.4 for 24 h, dehydrated in an ethanol series, and embedded in paraffin. Sections with a thickness of 5 μm were cut along the longitudinal axis, and one section from the middle level was used for the analysis. Sections were stained with H&E, safranin O and Masson’s trichrome.

Histologic scores were assigned by two blinded researchers, an experimented musculoskeletal pathologist and an orthopedic surgeon, according to the ICRS 1 score (Table 3) [49] and a new grading system designed to evaluate partial thickness chondral lesions (Table 4). The advantage of the ICRS 1 scale is that it is simple and frequently used in the literature. However, it was developed to evaluate biopsy samples in human patients; therefore, it has limitations in assessments of the whole defect and the borders of the lesion. Although not recommended by the authors who developed the ICRS 1 score [49], the use of the summed values of each sub-item as quantitative data is common in the literature. The sub-item Calcified Cartilage was excluded from the analysis because the calcified layer was preserved in this partial thickness model. The new scale is not yet validated and accepted in the literature. So it was not used as the primary outcome. A validation study is ongoing.

**Table 3:** The ICRS 1 histological scale.

	Feature	Score
I	Surface	
	Smooth/continuous	3
	Discontinuities/irregularities	0
II	Matrix	
	Hyaline	3
	Mixture: hyaline/fibrocartilage	2
	Fibrocartilage	1
	Fibrous tissue	0
III	Cell distribution	
	Columnar	3
	Mixed columnar/clusters	2
	Clusters	1
	Individual cells/ disorganized	0
IV	Cell viability	
	Predominantly viable	3
	Partially viable	1
	<10% viable	0
V	Subchondral bone	
	Normal	3
	Increased remodeling	2
	Bone necrosis/granulation tissue	1
	Detached/callus/fracture	0
VI	Calcified cartilage	
	Normal	3
	Abnormal/ inappropriate location	0

**Table 4:** The new Partial Thickness Model histological scale.

	Repair Tissue inside the Lesion	Feature	Score
I	Horizontal filling	75%–100%	4
		50%–74%	3
		20%–49%	2
		1%–19%	1
		0	0
II	Vertical filling	75%–100%	4
		50%–74%	3
		20%–49%	2
		1%–19%	1
		0	0
III	Cellularity	Normal	2
		Mild hypocellularity, <25% clusters	1
		Moderate or intense hypocellularity, >25% clusters	0
IV	Safranin staining	Homogeneous	2
		Heterogeneous	1
		Negative	0
	Cartilage Around the Lesion		
V	Borders ingrowth or integration	Bilateral	2
		Unilateral	1
		None	0
	Base of the Lesion		
VI	Residual calcified cartilage	Intact or integrated	3
		Fibrillation or fissure	2
		Focal erosion	1
		Severe disruption	0
VII	Subchondral bone	Normal	4
		Mild cystic lesions or granulation tissue	3
		Moderate or severe cystic lesions or granulation tissue	2
		Remodeling, sclerosis, callus	1
		Fracture, necrosis	0

### 4.8. Statistical Analysis

#### 4.8.1. Sample Size

Based on a previous study in which the standard deviation of the ICRS microscopic score was 0.7 with a power of 80% and a confidence level of 5%, a sample of 30 knees (10 in each group) was anticipated to be sufficient to detect a minimal clinically significant difference of 0.9 [51].

#### 4.8.2. Data Analysis

Quantitative data are expressed as the mean and standard deviation (SD). Qualitative data are expressed as the absolute frequency.

The Kolmogorov-Smirnov test was applied to continuous variables to determine whether the data followed a normal distribution.

Quantitative data were analyzed by one-way analysis of variance (ANOVA) followed by the Student-Newman-Keuls test and Dunnett’s test when parametrical assumptions could be fulfilled or by the Kruskal-Wallis test. Categorical data were analyzed with the Pearson Chi-Squared test or Fisher exact test. The significance level was set at 5%. All analyses were performed with IBM SPSS Statistics (Version 22.0. Armonk, IBM Corp., New York, NY, USA).

## 5. Conclusions

The regeneration of articular cartilage remains a challenge to the disciplines of Tissue Engineering and Regenerative Medicine. The use of cell-seeded scaffolds is one of the most promising strategies to solve this problem, but scaffold manufacturing and three-dimensional cell cultures techniques improvements, associated with advances in the understanding of native hyaline cartilage response to injuries and the molecular mechanisms by which MSCs could enhance it are still open questions in this field of knowledge. The paracrine effect of MSCs should be valued. The results of this study demonstrated that human AT-MSCs seeded onto collagen/chitosan scaffolds provided superior healing of partial thickness chondral lesions compared to the control groups, in an adult ovine model. This method needs to be improved, but seems to be an alternative to develop future treatment strategies suitable for human clinical trials, as adipose tissue offers an easy and efficient way to harvest MSCs.
